# Relationship between nutritional-inflammatory markers and postoperative outcomes in ovarian cancer: a retrospective study

**DOI:** 10.3389/fonc.2025.1531987

**Published:** 2025-03-11

**Authors:** Ying Zhang, Wen Xing, Xiaoyi Liang, Zhujuan Yang, Yun Ma, Ying Chen, Weipei Zhu

**Affiliations:** Department of Obstetrics and Gynecology, The Second Affiliated Hospital of Soochow University, Suzhou, Jiangsu, China

**Keywords:** nutritional inflammatory markers, PNI, NPS, ovarian cancer, prognosis

## Abstract

**Background:**

Elevated inflammatory markers are commonly linked to poor prognoses in cancer patients, while optimal nutritional status correlates with improved survival outcomes. This study aimed to explore the interplay between nutritional and inflammatory markers and their impact on postoperative outcomes in ovarian cancer patients through a retrospective analysis.

**Methods:**

Data were retrospectively retrieved from patients diagnosed with ovarian cancer who required surgical intervention at the Department of Obstetrics and Gynecology. Overall survival (OS) and cancer-specific survival (CSS) were monitored during follow-up. Kaplan-Meier survival curves were employed to assess OS and CSS across different patient cohorts, evaluating the prognostic significance of nutritional and inflammatory markers. Nomograms for predicting OS and CSS at one, three, and five years postoperatively were constructed, followed by external validation.

**Results:**

The prognostic nutritional index (PNI) and Naples prognostic score (NPS) exhibited a significant correlation with OS and CSS in postoperative ovarian cancer patients (*p <* 0.05). Analysis indicated that patients with a PNI > 51.2 demonstrated the most favorable survival outcomes. Furthermore, those with a low-NPS (L-NPS) had notably better survival rates compared to their high-NPS (H-NPS) counterparts. Independent OS predictors included age, PNI, NPS, histological type, tumor size, targeted therapy, and diabetes. Similarly, the CSS prediction model incorporated age, NPS, tumor size, targeted therapy, and diabetes. The nomograms demonstrated robust predictive accuracy for three- and five-year survival, though one-year calibration curves showed limited agreement. Despite slightly reduced external validation performance compared to the initial sample, the model maintained strong predictive capability.

**Conclusions:**

The nutritional inflammatory index serves as a key independent prognostic marker for OS and CSS in ovarian cancer patients. Nomograms based on PNI and NPS provide valuable prognostic insights for postoperative management. Incorporating these indices into clinical practice could improve patient stratification and guide personalized treatment plans.

## 1 Introduction

Ovarian cancer ranks among the three most prevalent gynecologic malignancies and remains a leading cause of mortality in this category. Its typically late-stage diagnosis, often resulting from the absence of distinct symptoms and the lack of early-stage screening, significantly compromises clinical outcomes (1, 2). Consequently, the efficacy of current treatments and the prognosis for ovarian cancer remain suboptimal. Modern therapeutic strategies must be informed by molecular profiling, as molecular biology plays a central role in shaping treatment paradigms (3). Increasing evidence underscores the complex interplay between nutrition, inflammation, and cancer prognosis, spurring ongoing investigations into these interactions across various malignancies, including ovarian cancer.

Several scoring systems that assess inflammation and nutritional status, including the lymphocyte-to-monocyte ratio (LMR), systemic inflammatory score (SIS), systemic immune-inflammatory index (SII), neutrophil-to-lymphocyte ratio (NLR), and COUNT, have demonstrated clinical relevance in gynecological cancers (4, 5). Among these, the prognostic nutritional index (PNI), Naples prognostic score (NPS), NLR, and LMR are particularly notable as nutritional-inflammation markers. PNI, initially introduced by Onodera (6), serves as a key indicator of both nutritional and inflammatory conditions, calculated from serum albumin levels and lymphocyte counts. Recent studies have revealed a strong correlation between PNI and prognosis in various cancers, as well as conditions like myocardial infarction and congenital heart disease (7, 8). Similarly, the NPS, developed in 2017 (9), integrates both inflammatory and nutritional biomarkers—albumin, total cholesterol (TC), NLR, and LMR—offering a comprehensive assessment of immune and nutritional status. The NPS has been validated as a reliable predictor of prognosis in multiple cancers, including colon, gallbladder, endometrial, and lung cancers (10–12). Emerging data emphasize the prognostic role of nutritional status in cancer patients, suggesting that markers like PNI and NPS may notably impact OS and cancer-specific survival (CSS) in ovarian cancer.

Elevated inflammatory markers often correlate with poor prognosis in cancer patients, while improved nutritional status is linked to enhanced survival outcomes. However, the specific influence of nutritional markers on ovarian cancer prognosis remains inadequately studied, revealing a notable research gap. This retrospective study seeks to explore the relationship between nutritional inflammatory markers and postoperative prognosis in ovarian cancer patients.

## 2 Methods

### 2.1 Study population

Between January 2022 and December 2023, this study enrolled 199 patients from the Department of Obstetrics and Gynecology at the Second People’s Hospital Affiliated to Suzhou University, along with 120 patients from the Department of Obstetrics and Gynecology at Binhai County People’s Hospital in Yancheng City, who were diagnosed with ovarian cancer and required surgical intervention. The study received approval and oversight from the Ethics Committee of the Second People’s Hospital Affiliated of Soochow University (Approval No. JD-LK-2022-079-01) and Binhai County People’s Hospital in Yancheng City (Approval No. 2024-BYKYLL-041). The study was conducted in accordance with the revised 2013 Helsinki Declaration. Written informed consent was obtained from each participant or their legal representative.

The inclusion criteria were as follows: (1) age ≥18 years; (2) a confirmed diagnosis of ovarian cancer requiring surgical intervention, as verified by a physician; and (3) provision of signed informed consent by the patient.

The exclusion criteria were as follows: (1) incomplete laboratory data; (2) a diagnosis of ovarian cancer without surgical treatment; and (3) refusal to provide informed consent.

### 2.2 Clinical data collection and follow-up

The study included a broad spectrum of variables: (1) Demographic data: age and body mass index (BMI); (2) Laboratory markers: TC, albumin, neutrophil, lymphocyte, and monocyte counts, carbohydrate antigen 125 (CA125), CA199, human epididymis secretory protein 4 (HE4), and carcinoembryonic antigen (CEA); (3) Comorbid conditions: hypertension and diabetes; (4) Tumor characteristics: tumor size, type of surgery (PDS or IDS), residual tumor post-surgery (yes or no), coronavirus disease 2019 (COVID-19) infection (yes or no), pathological type, tissue type, lymph node metastasis, International Federation of Gynecology and Obstetrics (FIGO) grade, differentiation status, history of chemotherapy, and targeted therapy; and (5) Length of hospital stay. Trained professionals collected laboratory markers and tumor characteristics. Biochemical analyses were conducted using a standardized automated system under the supervision of laboratory physicians, while pathologists determined tumor diagnoses and grading. Data on preexisting conditions were extracted from patients’ medical histories, and the hospital discharge system recorded the length of stay. Follow-up data were obtained through outpatient visits or telephone interviews. Survival outcomes were assessed at one, three, and five years post-surgery. The primary endpoints of the study were OS and CSS.

**Figure 1:**
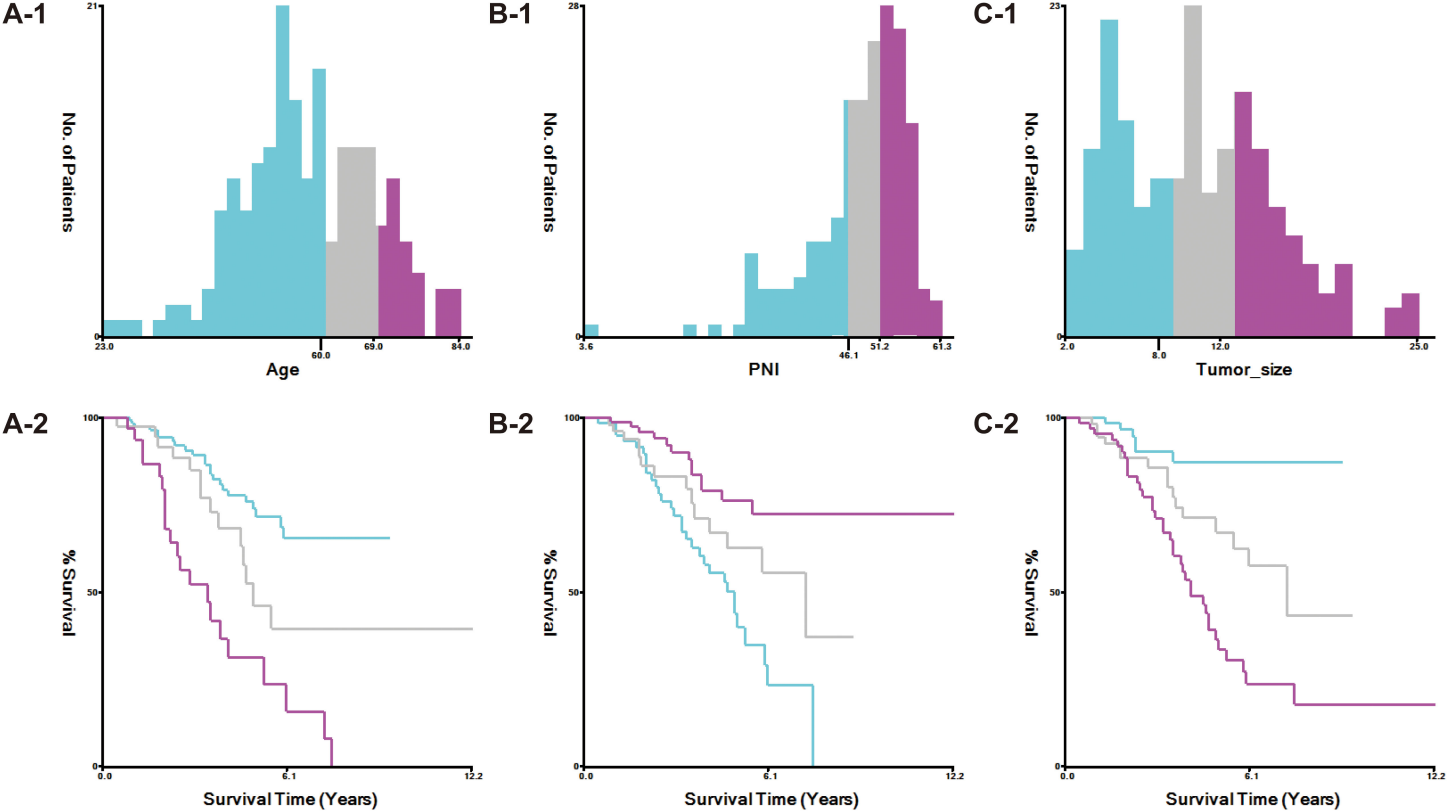
X-tile software defines age **(A)**, PNI **(B)**, and tumor size **(C)**.

The study stratified variables according to criteria established by X-tile software (**Figure 1**). Age was categorized into three groups: ≤59 years, 60–69 years, and ≥70 years. Tumor size was classified as <8 cm, 8–12 cm, and >12 cm.

### 2.3 Calculation of nutritional inflammatory markers

The nutritional inflammatory markers, NLR, LMR, PNI, and NPS, were calculated using the following formulas: (1) NLR as the ratio of neutrophil to lymphocyte counts; (2) LMR as the ratio of lymphocyte to monocyte counts; (3) PNI as albumin (g/L) plus five times the lymphocyte count; and (4) NPS, determined based on serum albumin, TC, NLR, and LMR levels following established methodologies. Using X-tile software, PNI was stratified into three categories: <46.1, 46.1–51.2, and >51.2 (**Figure 1**). NPS was assigned based on these criteria: (1) a score of 0 for serum albumin ≥40 g/L, TC >4.68 mmol/L, NLR <2.96, and LMR >4.44; (2) a score of 1 for serum albumin <40 g/L, TC ≤4.68 mmol/L, NLR ≥2.96, and LMR ≤4.44. The total NPS was calculated by summing the scores of these four parameters. Cumulative NPS values of 0–2 were categorized as low-NPS (L-NPS), while scores of 3–4 were defined as high-NPS (H-NPS).

### 2.4 Statistical analysis

The baseline characteristics of all enrolled patients were stratified according to survival outcomes. Non-normally distributed variables were expressed as quartile ranges and analyzed using the Wilcoxon rank-sum test. Categorical variables were presented as percentages and compared with chi-square tests. Univariate and multivariate Cox regression models were applied to calculate hazard ratios (HRs) and assess the independence of nutritional inflammatory indices as prognostic factors for OS and CSS in ovarian cancer patients. Kaplan-Meier curves were generated to illustrate OS and CSS across patient groups. To further examine the relationship between nutritional inflammatory markers and survival outcomes, restricted cubic spline (RCS) plots were constructed. A nomogram incorporating statistically significant markers from the multivariate Cox regression was developed. The nomogram’s diagnostic performance was evaluated using receiver operating characteristic (ROC) curves and calibration plots. ROC curves were also used to assess the prognostic value of different markers for ovarian cancer patients. External validation was performed concurrently. Finally, stratified analyses were conducted to explore the effects of various nutritional inflammatory indices on subpopulations of ovarian cancer patients. Statistical analyses were performed with R software (version 4.3.0) and STATA 17.0 (64-bit). A bilateral *p*-value <0.05 was considered statistically significant.

## 3 Results

### 3.1 Demographic and clinical characteristics of patients with ovarian cancer

A cohort of 199 ovarian cancer patients who underwent surgical treatment at the Second Affiliated Hospital of Suzhou University was analyzed. Of these, 141 patients (70.85%) survived, while 58 patients (29.15%) succumbed to the disease. The demographic and clinical characteristics of both groups were summarized in **Table 1**. Increased mortality risk was associated with advanced age, lower albumin levels, reduced lymphocyte counts, decreased PNI, elevated neutrophil counts, higher NLR, increased HE4, H-NPS, and postoperative tumor residue. Additional high-risk factors included high-grade histology, larger tumor size, FIGO stage IV, poor tumor differentiation, absence of targeted therapy, and a history of diabetes. Statistically significant differences between survivors and deceased patients were observed for all these variables (all *p <* 0.05). Furthermore, deceased patients had longer hospital stays compared to survivors. No significant differences were found regarding monocyte counts, CA125, CA199, CEA, tumor histological type, surgical procedure, COVID-19 infection, or lymphatic metastasis. The lack of statistical significance in postoperative survival and prognosis may be attributed to the small sample size of this study.

**Table 1:** Baseline demographic and clinical characteristics of patients with ovarian cancer.

Characteristic	Total No. (%)	The Second People’s Hospital Affiliated of Soochow University		P value	Total No. (%)	Binhai County People’s Hospital		P value
		Survival, No. (%)	Dead, No. (%)			Survival, No. (%)	Dead, No. (%)	
Total	199	141 (70.85)	58 (29.15)		120	86	34	
Age, years				**<0.001**				**< 0.001**
≤59	114 (57.3%)	94 (66.7%)	20 (34.5%)		65 (54.2%)	55 (45.8%)	10 (8.3%)	
60-69	52 (26.1%)	35 (24.8%)	17 (29.3%)		39 (32.5%)	26 (21.7%)	13 (10.8%)	
≥70	33 (16.6%)	12 (8.5%)	21 (36.2%)		16 (13.3%)	5 (4.2%)	11 (9.2%)	
BMI	24.64(22.41, 28.58)	24.27 (22.15, 27.70)	27.06 (23.63, 30.14)	**0.005**	25.28 (22.64, 28.80)	25.42 ± 3.84	28.08 ± 6.31	**0.027**
Albumin	42.8 (38, 45.9)	43.9 (39.5, 46.9)	39.65 (34.8, 44.2)	**< 0.001**	41.14 ± 4.60	42.17 ± 4.13	38.54 ± 4.77	**< 0.001**
Total cholesterol	4.2 ± 1.25	4.41 ± 1.20	4.05 ± 1.33	0.064	4.44 ± 1.05	4.48 ± 1.06	4.32 ± 1.02	0.435
Lymphocyte	1.3 (1, 1.6)	1.4 (1.1, 1.7)	1.2 (0.93, 1.5)	**0.010**	1.33 (1.1, 1.7)	1.4 (1.11, 1.79)	1.22 (1.06, 1.41)	**0.013**
Neutrophil	4.3 (3.3, 6)	4.2 (3.2, 5.8)	5 (3.73, 6.68)	**0.037**	4.24 (3.21, 6.32)	4.15 (3.26, 5.46)	4.86 (3.15, 8.18)	0.137
Monocyte	0.4 (0.3, 0.5)	0.4 (0.3, 0.5)	0.4 (0.3, 0.5)	0.688	0.37 (0.26, 0.56)	0.37 (0.25, 0.57)	0.40 (0.3, 0.54)	0.358
NLR	3.25 (2.21, 4.96)	2.88 (2.15, 4.63)	4.11 (2.72, 5.93)	**0.011**	3.12 (1.94, 4.87)	2.84 (1.94, 4.61)	4.39 (2.94, 5.52)	**0.031**
LMR	3.67 (2.33, 5.0)	3.75 (2.5, 5.0)	3 (2, 4.63)	0.093	3.41 (2.52, 5.0)	3.5 (2.64, 5.39)	3.27 (2.0, 4.0)	**0.055**
CA125	297 (62.73, 969)	295.5 (50.08, 920.25)	331.7 (100, 969)	0.438	348 (102, 625)	289 (67, 568)	316 (375, 604)	0.211
CA199	12.08 (6.56, 23)	10.65 (6.40, 23.35)	13.92 (7.03, 22.8)	0.416	23.27 (11.86, 93.84)	22.38 (12.38, 83.84)	24.10 (11.07, 210.88)	0.606
CEA	1.4 (0.86, 2.33)	1.385 (0.86, 2.29)	1.56 (0.92, 2.62)	0.710	3.4 (2.18, 7.85)	2.97 (2.14, 6.08)	3.07 (2.34, 9.55)	0.088
HE4	167 (73.67, 458.2)	134.5 (67, 367.77)	202.6 (111.78, 536.38)	**0.042**	269.7 (149.41, 412.2)	245.75 (121.18, 406.05)	313.5 (252.45, 431.2)	**0.020**
PNI, n (%)				**< 0.001**				**< 0.001**
<46.1	61 (30.7%)	31 (22.0%)	30 (51.7%)		39 (32.5%)	20 (16.7%)	19 (15.8%)	
46.1-51.2	57 (28.6%)	42 (29.8%)	15 (25.9%)		44 (36.7%)	33 (27.5%)	11 (9.2%)	
>51.2	81 (40.7%)	68 (48.2%)	13 (22.4%)		37 (30.8%)	33 (27.5%)	4 (3.3%)	
NPS, n (%)				**0.003**				**< 0.001**
L-NPS	101 (50.8%)	81 (57.4%)	20 (34.5%)		77 (64.2%)	64 (53.3%)	13 (10.8%)	
H-NPS	98 (49.2%)	60 (42.6%)	38 (65.5%)		43 (35.8%)	22 (18.3%)	21 (17.5%)	
Pathological type, n (%)				0.362				0.087
Serous adenocarcinoma	140 (70.4%)	95 (67.4%)	45 (77.6%)		81 (67.5%)	54 (45%)	27 (22.5%)	
Endometrioid adenocarcinoma	28 (14.1%)	20 (14.2%)	8 (13.8%)		17 (14.2%)	16 (13.3%)	1 (0.8%)	
Mucinous adenocarcinoma	14 (7%)	12 (8.5%)	2 (3.4%)		14 (11.7%)	9 (7.5%)	5 (4.2%)	
Clear cell carcinoma	17 (8.5%)	14 (9.9%)	3 (5.2%)		8 (6.7%)	7 (5.8%)	1 (0.8%)	
Histology type, n (%)				0.021				0.064
High-grade	168 (84.4%)	113 (80.1%)	55 (94.8%)		91 (75.8%)	62 (51.7%)	29 (24.2%)	
Medium-grade	9 (4.5%)	7 (5%)	2 (3.4%)		11 (9.2%)	7 (5.8%)	4 (3.3%)	
Low-grade	22 (11.1%)	21 (14.9%)	1 (1.7%)		18 (15%)	17 (14.2%)	1 (0.8%)	
Lymph node metastasis, n (%)				0.076				**< 0.001**
No	95 (47.7%)	73 (51.8%)	22 (37.9%)		57 (47.5%)	50 (41.7%)	7 (5.8%)	
Yes	104 (52.3%)	68 (48.2%)	36 (62.1%)		63 (52.5%)	36 (30%)	27 (22.5%)	
Tumor size				**<0.001**				0.295
<8	65 (32.7%)	59 (41.8%)	6 (10.3%)		34 (28.3%)	27 (22.5%)	7 (5.8%)	
8-12	68 (34.2%)	52 (36.9%)	16 (27.6%)		45 (37.5%	33 (27.5%)	12 (10%)	
>12	66 (33.2%)	30 (21.3%)	36 (62.1%)		41 (34.2%)	26 (21.7%)	15 (12.5%)	
FIGO stage, n (%)				0.055				**< 0.001**
Grade I	47 (23.6%)	40 (28.4%)	7 (12.1%)		28 (23.3%)	28 (23.3%)	0 (0%)	
Grade II	27 (13.6%)	19 (13.5%)	8 (13.8%)		16 (13.3%)	13 (10.8%)	3 (2.5%)	
Grade III	105 (52.8%)	71 (50.4%)	34 (58.6%)		61 (50.8%)	40 (33.3%)	21 (17.5%)	
Grade IV	20 (10.1%)	11 (7.8%)	9 (15.5%)		15 (12.5%)	5 (4.2%)	10 (8.3%)	
Degree of differentiation, n (%)				**0.007**				**0.005**
Low-differentiated	167 (83.9%)	111 (78.7%)	56 (96.6%)		98 (81.7%)	64 (53.3%)	34 (28.3%)	
Medium-differentiated	9 (4.5%)	8 (5.7%)	1 (1.7%)		6 (5%)	6 (5%)	0 (0%)	
High-differentiated	23 (11.6%)	22 (15.6%)	1 (1.7%)		16 (13.3%)	16 (13.3%)	0 (0%)	
Chemotherapy, n (%)				0.569				**0.007**
No	21 (10.6%)	16 (11.3%)	5 (8.6%)		17 (14.2%)	7 (5.8%)	10 (8.3%)	
Yes	178 (89.4%)	125 (88.7%)	53 (91.4%)		103 (85.8%)	79 (65.8%)	24 (20%)	
Targeted therapy, n (%)				**< 0.001**				**< 0.001**
No	128 (64.3%)	74 (52.5%)	54 (93.1%)		91 (75.8%)	57 (47.5%)	34 (28.3%)	
Yes	71 (35.7%)	67 (47.5%)	4 (6.9%)		29 (24.2%)	29 (24.2%)	0 (0%)	
Type of surgery, n (%)				0.085				**0.030**
PDS	191 (96.0%)	138 (97.9%)	53 (91.4%)		113 (94.2%)	84 (70%)	29 (24.2%)	
IDS	8 (4.0%)	3 (2.1%)	5 (8.6%)		7 (5.8%)	2 (1.7%)	5 (4.2%)	
Residual tumoral surgery, n (%)				<0.001				**<0.001**
No	175 (87.9%)	134 (95.0%)	41 (70.7%)		86 (71.7%)	79 (65.8%)	7 (5.8%)	
Yes	24 (12.1%)	7 (5.0%)	17 (29.3%)		34 (28.3%)	7 (5.8%)	27 (22.5%)	
COVD-19 infection				0.385				0.185
No	181 (90.95%)	133 (94.33%)	48 (82.76%)		99 (82.5%)	70 (58.33%)	29 (24.16%)	
Yes	18 (9.05%)	8 (5.67%)	10 (17.24%)		21 (17.5%)	16 (13.33%)	5 (4.17%)	
Hospital time	16 (14, 21)	16 (13, 20)	18.5 (14, 23)	**0.036**	18 (14, 23)	16 (14, 20)	21 (16.25, 24)	**0.032**
Hypertension, n (%)				0.583				0.506
No	136 (68.3%)	98 (69.5%)	38 (65.5%)		83 (69.2%)	61 (50.8%)	22 (18.3%)	
Yes	63 (31.7%)	43 (30.5%)	20 (34.5%)		37 (30.8%)	25 (20.8%)	12 (10%)	
Diabetes, n (%)				**< 0.001**				**< 0.001**
No	170 (85.4%)	129 (91.5%)	41 (70.7%)		103 (85.8%)	81 (67.5%)	22 (18.3%)	
Yes	29 (14.6%)	12 (8.5%)	17 (29.3%)		17 (14.2%)	5 (4.2%)	12 (10%)	

Percentages may not total 100 because of rounding. Bold values indicates p<0.05.

An analysis of 120 patients from Binhai County People’s Hospital (**Table 1**) yielded results consistent with those from the Second Affiliated Hospital to Suzhou University. Factors including advanced age, elevated BMI, low albumin levels, reduced lymphocyte count, increased NLR, elevated HE4, low PNI, H-NPS, lymphocyte metastasis, poor differentiation, absence of radiotherapy or targeted therapy, residual tumor post-surgery, and comorbid diabetes were all significantly associated with a higher risk of mortality (all *p<*0.05). In contrast, neutrophil levels did not exhibit statistical significance in the Binhai County cohort, which may be attributed to the smaller sample size in our study.

### 3.2 Identification of prognostic factors affecting OS and CSS in ovarian cancer postoperatively

**Figure 2:**
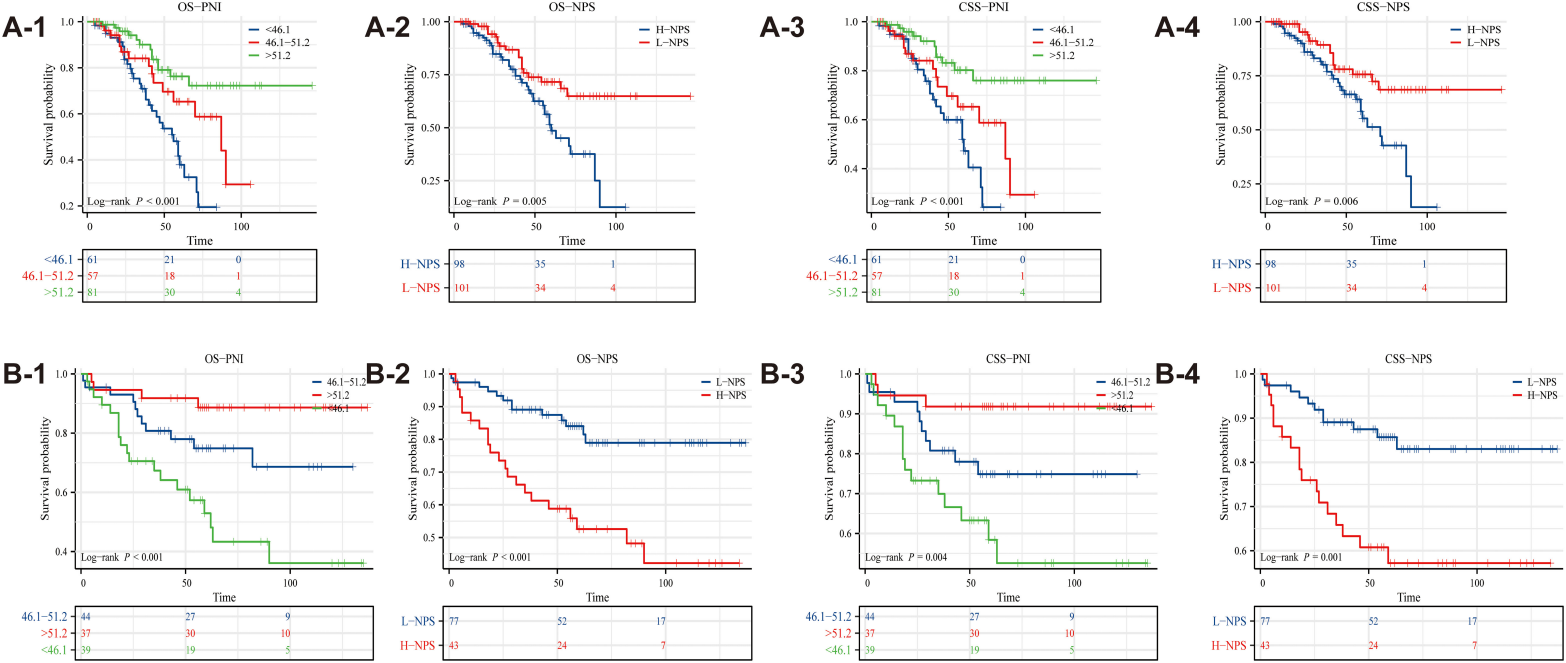
Kaplan-Meier survival curves evaluated the effects of PNI and NPS on survival outcomes (**A1-A4**: The Second People’s Hospital Affiliated of Soochow University; **B1-B4**: Binhai County People's Hospital).

**Figure 3:**
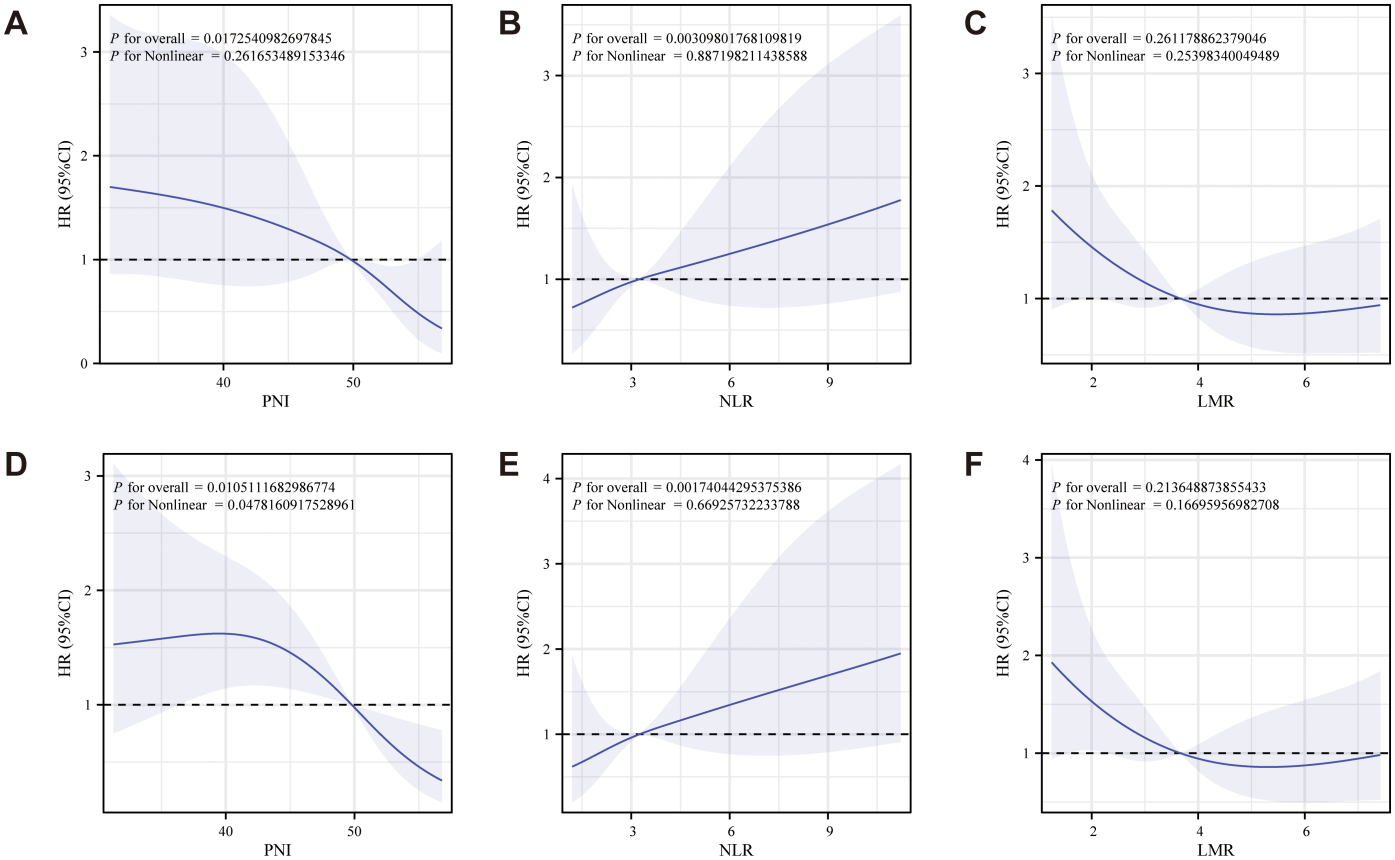
RCS diagram of the relationship between PNI, NLR, LMR and survival prognosis of patients with ovarian cancer after surgery (**A-C**: OS analysis showed that PNI and LMR were negatively correlated with prognosis, while NLR was positively correlated with prognosis; **D-F**: The CSS analysis showed that PNI and LMR were negatively associated with prognosis, while NLR remained positively associated).

**Figure 4:**
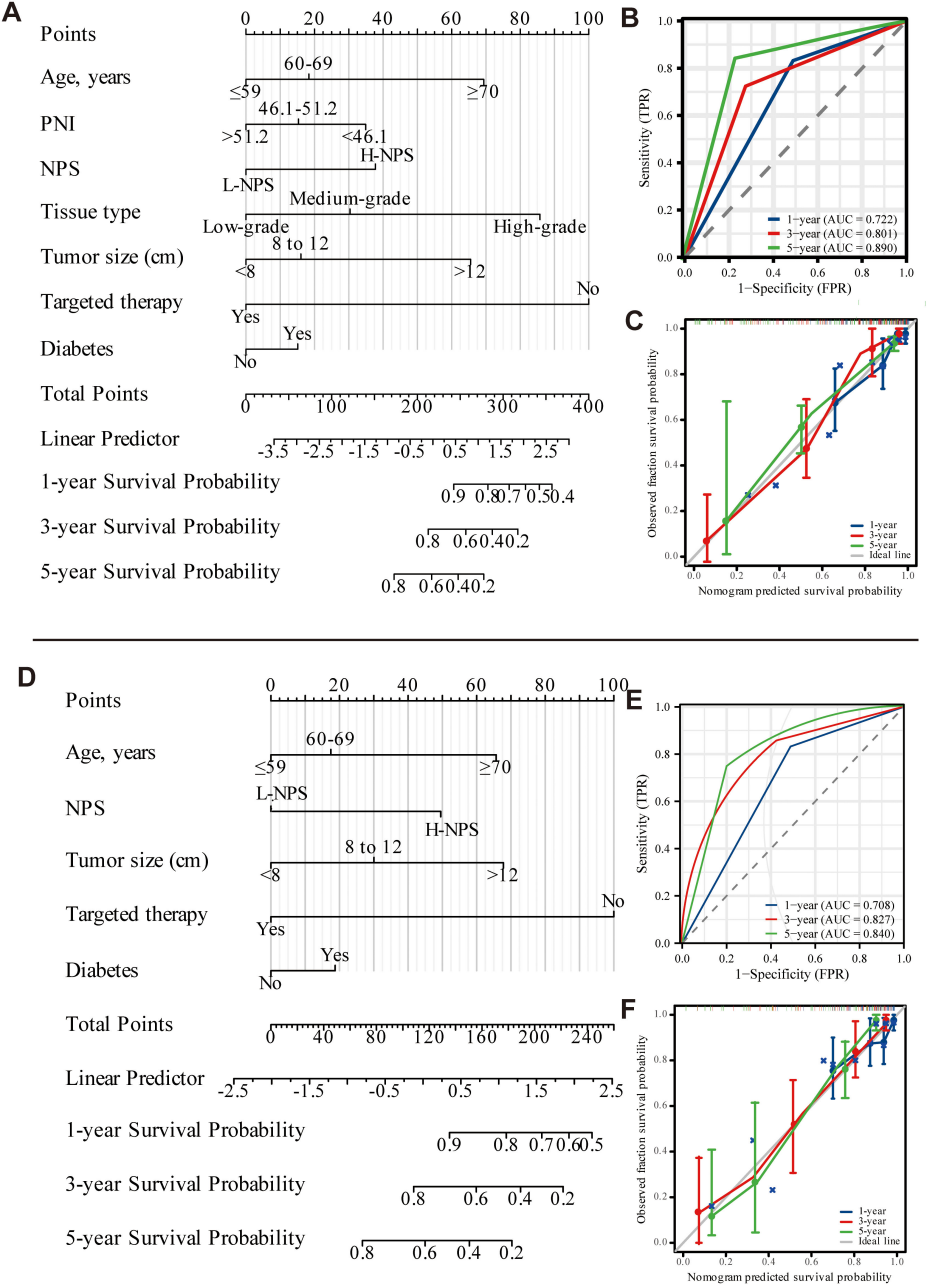
Nomogram of OS and CSS 1, 3, and 5 years after ovarian cancer surgery (**A-C**: OS; **D-F**: CSS).

Kaplan-Meier survival analysis revealed significant associations between PNI, NPS, and survival outcomes in ovarian cancer patients (*p <* 0.05), as depicted in **Figure 2**. Patients with PNI > 51.2 exhibited the best OS, whereas those with PNI < 46.1 had the poorest prognosis (**Figures 2A-1**). Likewise, survival outcomes were significantly better for patients with L-NPS compared to those with H-NPS (**Figure 2A-2**). These patterns were similarly observed for CSS, as shown in **Figures 2A-3, 2A-4**. Consistent results were obtained from the data of Binhai County People’s Hospital (**Figures 2B-1–4**).

Univariate and multivariate Cox regression analyses were conducted to identify factors influencing OS and CSS. For OS, independent risk factors included age, PNI, NPS, tissue type, tumor size, targeted therapy status, and diabetes (**Table 2**). Specifically, patients with PNI between 46.1 and 51.2 demonstrated improved survival outcomes compared to those with PNI <46.1 (HR = 0.461; 95% CI, 0.234–0.864; *p =* 0.036), while those with PNI >51.2 exhibited even more pronounced protective effects (HR = 0.357; 95% CI, 0.095–0.538; *p =* 0.025). In contrast, patients with H-NPS had significantly worse survival compared to those with L-NPS (HR = 1.576; 95% CI, 1.252–2.719; *p =* 0.013). For CSS, multivariate Cox regression analysis (**Table 3**) identified age, NPS, tumor size, targeted therapy status, and diabetes as independent risk factors. Consistent with OS, patients with H-NPS experienced poorer outcomes compared to those with L-NPS (HR = 1.654; 95% CI, 1.278–3.138; *p =* 0.031).

**Table 2:** Univariate analysis of overall survival (OS) in patients with ovarian cancer.

Characteristic	OS			
	Univariate analysis	Multivariate analysis		
	Hazard Ratio (95% CI)	*P* value	Hazard Ratio (95% CI)	*P* value
Age, years				
≤59	Reference		Reference	
60-69	1.852 (0.969,3.542)	0.062	1.223 (0.583,2.564)	0.594
≥70	5.158 (2.784,9.556)	**<0.001**	**3.320 (1.565,7.046)**	**0.002**
BMI	1.006 (0.963,1.050)	0.801	–	–
Albumin	0.951 (0.922,0.981)	**0.001**	1.052 (0.978,1.132)	0.170
Total cholesterol	0.920 (0.751,1.128)	0.423	–	–
Lymphocyte	0.671 (0.364,1.235)	0.200	–	–
Neutrophil	1.157 (1.073,1.248)	**< 0.001**	1.000 (0.798,1.254)	0.999
Monocyte	1.460 (0.870,2.448)	0.152	–	–
NLR	1.071 (1.031,1.113)	**< 0.001**	1.038 (0.940,1.146)	0.459
LMR	0.920 (0.799,1.059)	0.245	–	–
CA125	1.000 (1.000,1.000)	0.461	–	–
CA199	1.000 (0.999,1.001)	0.679	–	–
CEA	1.002 (1.000,1.004)	0.087	–	–
HE4	1.001 (1.000,1.001)	0.103	–	–
PNI, n (%)				
<46.1	Reference		Reference	
46.1-51.2	0.517 (0.276,0.970)	**0.040**	**0.461 (0.234,0.864)**	**0.036**
>51.2	0.259 (0.133,0.503)	**<0.001**	**0.357 (0.095,0.538)**	**0.025**
NPS, n (%)				
L-NPS	Reference		Reference	
H-NPS	2.139 (1.238,3.696)	**0.006**	**1.576 (1.252,2.719)**	**0.013**
Pathological type, n (%)				
Serous adenocarcinoma	Reference		–	–
Endometrioid adenocarcinoma	0.843 (0.397,1.790)	0.657	–	–
Mucinous adenocarcinoma	0.410 (0.099,1.691)	0.217	–	–
Clear cell carcinoma	0.576 (0.179,1.854)	0.355	–	–
Histology type, n (%)				
Low-grade	Reference		Reference	
Medium-grade	3.078 (0.551,6.091)	0.141	3.086 (0.551,6.183)	0.140
High-grade	5.321 (1.285,10.635)	**0.027**	**5.060 (1.106,10.722)**	**0.039**
Lymph node metastasis, n (%)				
No	Reference		Reference	
Yes	1.912 (1.123,3.258)	**0.017**	1.124 (0.466,2.709)	0.795
Tumor size				
<8	Reference		Reference	
8-12	2.412 (0.943,6.167)	0.066	1.502 (0.509,4.432)	0.461
>12	5.799 (2.443,13.769)	**<0.001**	**5.011 (1.858,13.511)**	**0.001**
FIGO stage, n (%)				
Grade I	Reference		Reference	
Grade II	2.544 (0.907,7.135)	0.076	0.698 (0.177,2.753)	0.608
Grade III	3.024 (1.325,6.902)	**0.009**	1.432 (0.412,4.975)	0.572
Grade IV	4.357 (2.633,9.558)	**<0.001**	1.860 (0.387,8.946)	0.439
Degree of differentiation, n (%)				
Low-differentiated	Reference		Reference	
Medium-differentiated	0.238 (0.033,1.734)	0.157	0.060 (0.003,1.420)	0.081
High-differentiated	0.099 (0.014,0.722)	**0.022**	0.299 (0.074,1.722)	0.997
Chemotherapy, n (%)				
No	Reference		-	-
Yes	0.635 (0.252,1.600)	0.336	-	-
Targeted therapy, n (%)				
No	Reference		Reference	
Yes	0.111 (0.040,0.309)	**<0.001**	**0.158 (0.048,0.516)**	**0.002**
Type of surgery, n (%)				
IDS	Reference		Reference	0.391
PDS	0.338 (0.134,0.851)	**0.021**	0.530 (0.124,2.262)	
Residual tumoral surgery, n (%)				
No	Reference		Reference	
Yes	3.199 (1.813,5.643)	**<0.001**	2.106 (0.895,4.956)	0.088
COVID-19 infection				
No	Reference			
Yes	1.112 (0.823,1.658)	0.321		
Hospital time	1.029 (0.995 - 1.064)	0.096	-	-
Hypertension, n (%)				
No	Reference		-	-
Yes	1.545 (0.897,2.660)	0.117	-	-
Diabetes, n (%)				
No	Reference		Reference	
Yes	2.923 (1.654,5.165)	**<0.001**	**1.603 (1.124,2.921)**	**0.041**

OS, Overall survival. Bold values indicates p<0.05.

**Table 3:** Univariate analysis of cancer-specific survival (CSS) in patients with ovarian cancer.

Characteristic	CSS			
	Univariate analysis	Multivariate analysis		
	Hazard Ratio (95% CI)	*P* value	Hazard Ratio (95% CI)	*P* value
Age, years				
≤59	Reference		Reference	
60-69	1.602 (0.801,3.201)	0.182	1.116 (0.506,2.459)	0.786
≥70	4.354 (2.253,8.415)	**<0.001**	**2.979 (1.346,6.596)**	**0.007**
BMI	1.009 (0.964,1.056)	0.711	–	–
Albumin	0.957 (0.925,0.990)	**0.011**	1.067 (0.982,1.159)	0.126
Total cholesterol	0.962 (0.774,1.196)	0.725	–	–
Lymphocyte	0.571 (0.293,1.113)	0.100	–	–
Neutrophil	1.174 (1.084,1.271)	**<0.001**	0.995 (0.789,1.256)	0.969
Monocyte	1.507 (0.886,2.562)	0.130	–	–
NLR	1.074 (1.030,1.120)	**<0.001**	1.055 (0.957,1.163)	0.280
LMR	0.922 (0.792,1.072)	0.290	–	–
CA125	1.002 (1.000,1.004)	0.314	–	–
CA199	1.000 (0.998,1.001)	0.596	–	–
CEA	1.002 (1.000,1.004)	0.058	–	–
HE4	1.001 (1.000,1.001)	0.072	–	–
PNI, n (%)				
<46.1	Reference		Reference	
46.1-51.2	0.633 (0.328,1.220)	0.172	0.690 (0.236,2.022)	0.499
>51.2	0.268 (0.129,0.555)	**<0.001**	0.321 (0.077,1.336)	0.118
NPS, n (%)				
L-NPS	Reference		Reference	
H-NPS	2.230 (1.234,4.030)	**0.008**	**1.654 (1.278,3.138)**	**0.031**
Pathological type, n (%)				
Serous adenocarcinoma	Reference		–	–
Endometrioid adenocarcinoma	0.865 (0.386,1.936)	0.723	–	–
Mucinous adenocarcinoma	0.478 (0.115,1.982)	0.309	–	–
Clear cell carcinoma	0.439 (0.106,1.819)	0.256	–	–
Histology type, n (%)				
Low-grade	Reference		Reference	
Medium-grade	3.086 (0.551,6.183)	0.140	2.086 (0.351,4.183)	0.540
High-grade	5.060 (1.106,10.722)	**0.039**	3.060 (0.706,5.722)	0.340
Lymph node metastasis, n (%)				
No	Reference		Reference	
Yes	1.905 (1.073,3.381)	**0.028**	1.167 (0.442,3.085)	0.755
Tumor size				
<8	Reference		Reference	
8-12	2.713 (0.985,7.470)	0.053	1.680 (0.561,5.030)	0.354
>12	5.789 (2.245,14.927)	**<0.001**	**4.163 (1.529,8.333)**	**0.005**
FIGO stage, n (%)				
Grade I	Reference		Reference	
Grade II	3.104 (1.052,9.159)	**0.040**	0.933 (0.220,3.959)	0.925
Grade III	2.886 (1.174,7.092)	**0.021**	1.372 (0.346,5.444)	0.653
Grade IV	4.899 (3.012,8.286)	**<0.001**	2.671 (0.514,13.868)	0.242
Degree of differentiation, n (%)				
Low-differentiated	Reference		Reference	
Medium-differentiated	0.272 (0.037,1.993)	0.200	0.229 (0.026,2.021)	0.185
High-differentiated	0.114 (0.016,0.835)	**0.032**	0.222 (0.026,1.878)	0.167
Chemotherapy, n (%)				
No	Reference		-	-
Yes	0.545 (0.215,1.383)	0.201	-	-
Targeted therapy, n (%)				
No	Reference		Reference	
Yes	0.129 (0.046,0.361)	**<0.001**	**0.204 (0.062,0.671)**	**0.009**
Type of surgery, n (%)				
IDS	Reference		Reference	0.469
PDS	0.288 (0.114,0.732)	**0.009**	0.585 (0.137,2.495)	
Residual tumoral surgery, n (%)				
No	Reference		Reference	
Yes	2.988 (1.608,5.552)	**<0.001**	1.537 (0.588,4.015)	0.381
COVID-19 infection				
No	Reference			
Yes	1.232 (0.873,1.418)	0.221		
Hospital time	1.031 (0.995,1.068)	0.089	-	-
Hypertension, n (%)				
No	Reference		-	-
Yes	1.372 (0.756,2.491)	0.298	-	-
Diabetes, n (%)				
No	Reference		Reference	
Yes	2.739 (1.471,5.100)	**0.001**	**2.016 (1.459,2.209)**	**0.024**

OS, Overall survival. Bold values indicates p<0.05.

### 3.3 Relationship between nutritional inflammatory markers and survival prognosis of postoperative patients with ovarian cancer

An RCS diagram (**Figure 3**) was constructed to explore the relationship between nutritional inflammatory markers and survival prognosis in postoperative ovarian cancer patients. OS analysis indicated a negative correlation between PNI and LMR with patient prognosis, suggesting that higher values of these indices were associated with improved survival outcomes (**Figures 3A, C**). In contrast, NLR exhibited a positive correlation with prognosis, with elevated NLR values linked to poorer survival (**Figure 3B**). The CSS analysis mirrored the findings from OS, revealing a negative correlation between both PNI and LMR with prognosis (**Figures 3D, F**), while NLR remained positively correlated, indicating worse outcomes as its value increased (**Figure 3E**). Additional analyses included markers such as albumin, TC, CA125, CA199, CEA, and HE4 (**Supplementary Figure S1**).

### 3.4 Construction and evaluation of the nomogram

Univariate and multivariate Cox regression analyses identified seven independent predictors of OS: age, PNI, NPS, tissue type, tumor size, targeted therapy use, and diabetes presence (*p <* 0.05; **Table 2**). Similarly, five variables were determined to be independent predictors of CSS (*p <* 0.05; **Table 3**). Nomograms incorporating these predictors were developed to estimate one-, three-, and five-year OS and CSS (**Figures 4A, D**). Risk scores for each factor in the nomogram, shown in **Figure 4**, indicated that higher scores corresponded to increased mortality risk. The predictive accuracy of the nomograms was assessed, yielding a concordance (C-index) of 0.715 for the OS nomogram and 0.704 for the CSS nomogram. ROC curves for one-, three-, and five-year OS and CSS were plotted, with the area under the curve (AUC) values for OS being 0.722, 0.801, and 0.890, respectively (**Figure 4B**). For CSS, the AUC values were 0.708, 0.827, and 0.840, respectively (**Figure 4E**). Calibration curves for three- and five-year predictions closely aligned with the diagonal, indicating high consistency and reliable calibration, whereas the one-year calibration curves deviated, suggesting lower robustness in predicting one-year outcomes (**Figures 4C, F**).

**Figure 5:**
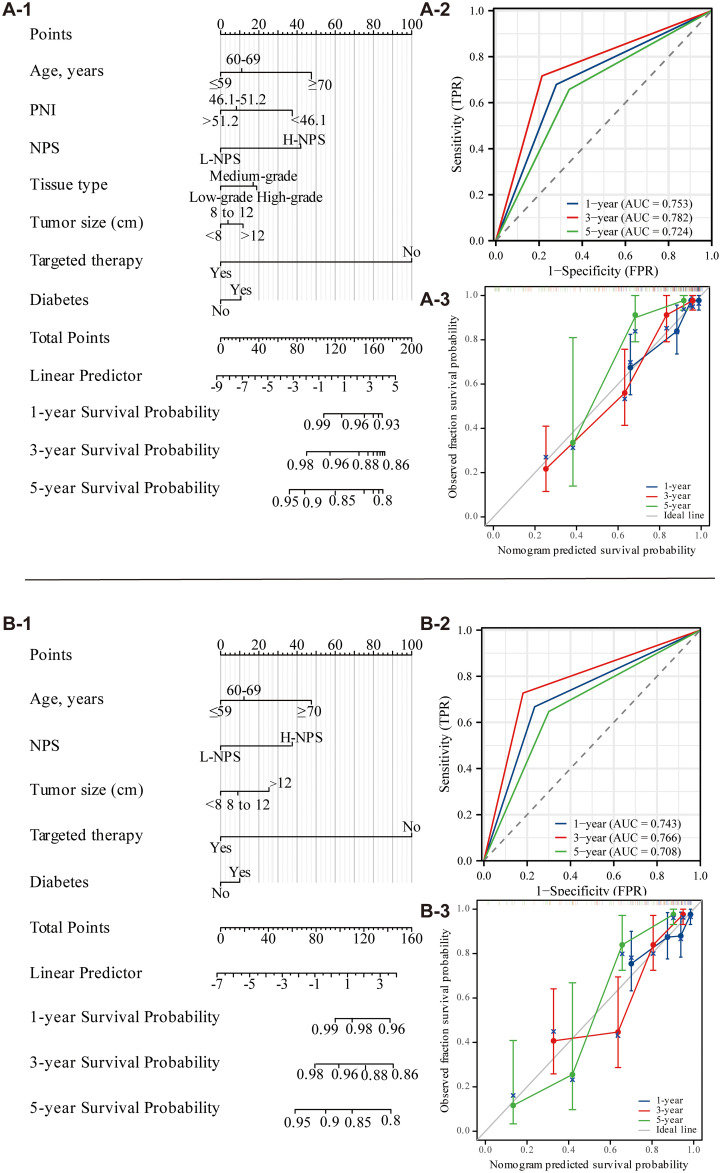
Nomogram of OS and CSS 1, 3, and 5 years after ovarian cancer surgery (external validation; **A**: OS; **B**: CSS).

External validation was performed using data from 120 patients at Binhai County People’s Hospital, applying the same characteristic variables to construct a nomogram for predicting 1-, 3-, and 5-year OS and CSS (**Figures 5A, B**). The nomogram’s risk scores for each factor were presented in **Figure 5**, where higher scores correlated with an increased risk of mortality. Predictive accuracy was assessed by evaluating the C-index, which was 0.735 for the OS nomogram and 0.714 for the CSS nomogram. ROC curves for 1-year, 3-year, and 5-year OS and CSS were also generated. For OS, AUC values were 0.753, 0.782, and 0.724, respectively (**Figure 5A-2**), while for CSS, AUC values were 0.743, 0.766, and 0.708 (**Figure 5B-2**). Calibration curves for 3- and 5-year predictions closely approximated the diagonal, reflecting strong consistency and reliable calibration. In contrast, the 1-year calibration curve showed notable deviation from the diagonal, indicating reduced predictive accuracy for the 1-year outcome (**Figures 5A-3, 5B-3**). A comparison with data from the Second People’s Hospital of Suzhou University revealed that while model performance at Binhai County People’s Hospital was somewhat lower, the evaluation metrics still confirmed the model’s overall effectiveness.

### 3.5 Predictive value of nutritional inflammatory markers for survival and prognosis of postoperative patients with ovarian cancer

**Figure 6:**
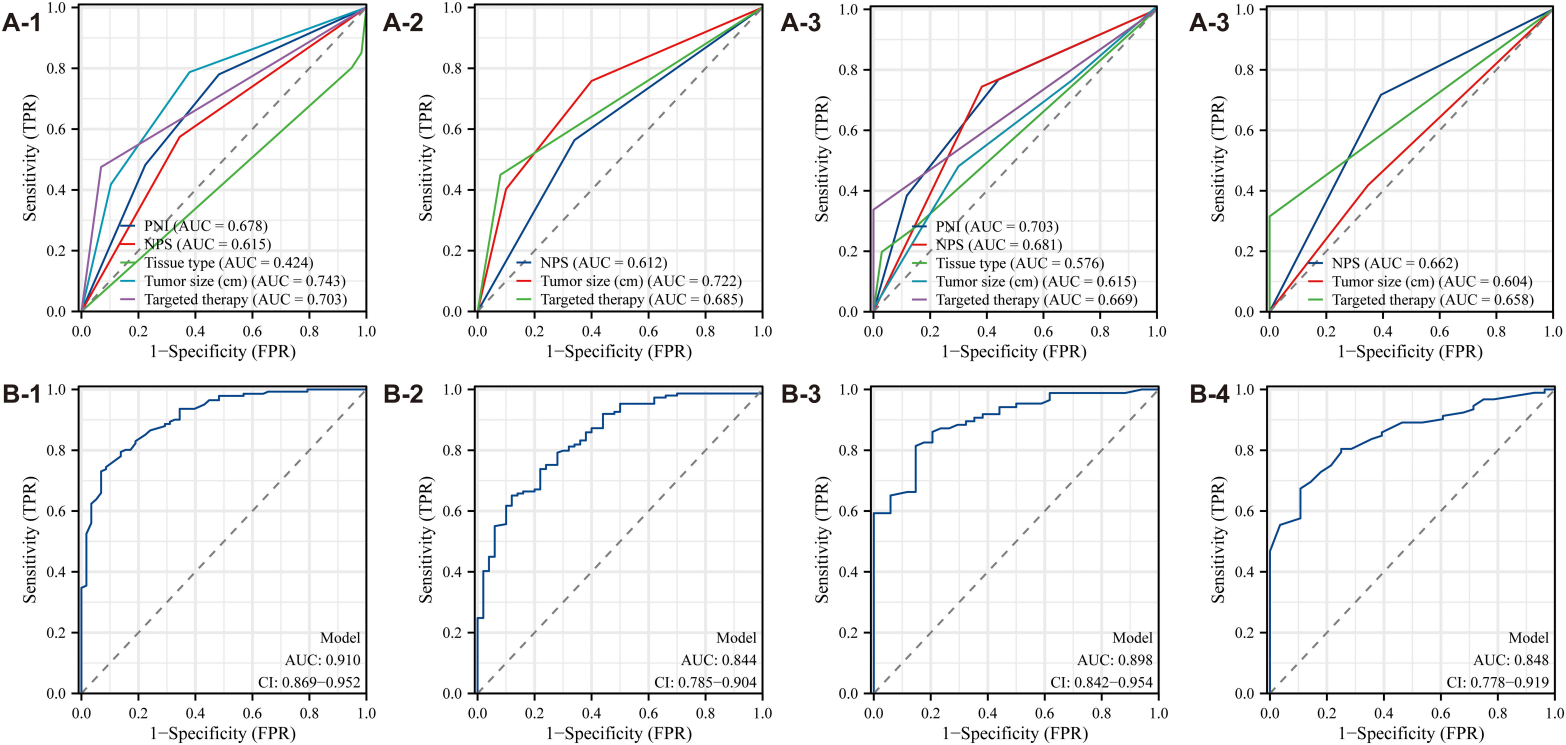
ROC curve of the diagnostic nomogram model (**A1-A4**: The Second People’s Hospital Affiliated of Soochow University; **B1-B4**: Binhai County People's Hospital).

Multivariate Cox regression analysis was used to develop a diagnostic nomogram, with correlated variables plotted alongside the ROC curve to evaluate its predictive accuracy for survival and prognosis in postoperative ovarian cancer patients (**Figure 6**). For OS, the model exhibited the highest predictive value, yielding an AUC of 0.910 (**Figure 6B-1**). In comparison, the AUCs for individual variables were 0.678 for PNI, 0.627 for NPS, 0.424 for tissue type, 0.743 for tumor size, and 0.703 for targeted therapy (**Figure 6A-1**). In the CSS analysis, the model again demonstrated the highest predictive value, achieving an AUC of 0.844 (**Figure 6B-2**). The AUCs for other variables were 0.612 for NPS, 0.722 for tumor size, and 0.685 for targeted therapy (**Figure 6A-2**). These results confirm the nomogram’s significant diagnostic utility, highlighting its capacity to accurately predict survival and prognosis in postoperative ovarian cancer patients.

Data analysis from Binhai County People’s Hospital revealed that the OS model exhibited the highest predictive value, with an AUC of 0.898 (**Figure 6B-3**). In contrast, the AUC values for other variables were as follows: PNI, 0.703; NPS, 0.681; tissue type, 0.576; tumor size, 0.618; and targeted therapy, 0.669 (**Figure 6A-3**). Likewise, for CSS, the model again demonstrated the highest predictive value, with an AUC of 0.848 (**Figure 6B-4**). The AUC values for additional variables were: NPS, 0.662; tumor size, 0.604; and targeted therapy, 0.658 (**Figure 6A-4**). These results further substantiate the diagnostic utility of the nomogram, reinforcing its robust predictive capability for survival and prognosis in postoperative ovarian cancer patients.

### 3.6 Subgroup analysis

**Figure 7:**
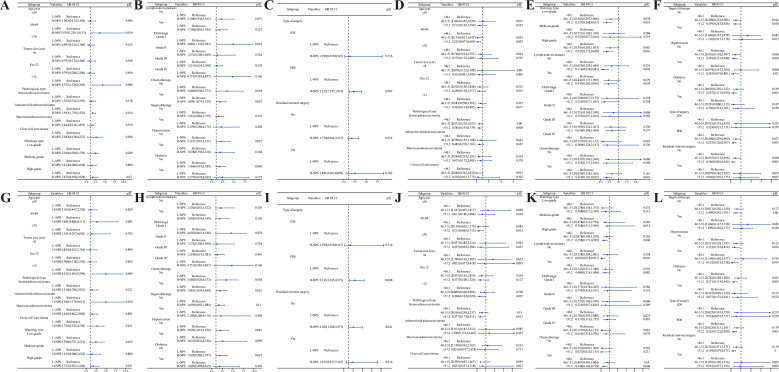
Subgroup analysis (**A-F**: OS; **G-L**: CSS).

Subgroup analysis of OS and CSS in postoperative ovarian cancer patients was performed based on factors such as age, tumor size, histological type, and tissue characteristics to assess survival prognosis. OS analysis using the NPS (**Figures 7A–C**) revealed that patients aged 60–69 years with H-NPS faced a significantly higher mortality risk than those with L-NPS (HR = 3.534; 95% CI, 1.230–10.151; *p =* 0.019). Similar patterns were observed in patients with tumors >12 cm, endometrioid adenocarcinoma, FIGO grade I, as well as those undergoing surgery and chemotherapy (all *p <* 0.05). Additionally, among patients aged 60–69 years, those with tumors >12 cm, serous adenocarcinoma, low-grade histology, no lymphatic metastasis, absence of targeted therapy, and without hypertension or diabetes, demonstrated the lowest mortality when PNI >51.2 (*p <* 0.05) (**Figures 7D–F**). In terms of CSS, the NPS analysis yielded comparable results (**Figures 7G–I**), with higher mortality rates in patients with tumors >12 cm and endometrioid adenocarcinoma, regardless of hypertension, surgical procedure, or residual tumor presence (all *p <* 0.05). Further PNI analysis for CSS (**Figures 7J–L**) indicated that patients aged 60–69 years with tumors >12 cm, serous adenocarcinoma, high-grade histology, absence of lymphatic metastasis, receiving chemotherapy, without targeted therapy, and regardless of hypertension or diabetes, surgery type, or residual tumor showed the lowest mortality rates when PNI >51.2 (all *p <* 0.05).

## 4 Discussion

Ovarian cancer ranks among the three most prevalent gynecologic malignancies and poses a significant public health threat. As the deadliest of all gynecologic cancers, it is characterized by a notably high mortality rate (13). This poor prognosis is largely due to late-stage diagnoses, as most patients present with advanced disease at the time of detection (1). Key factors, including age, tumor size, histological subtype, and comorbidities, significantly impact survival outcomes (14). The advent of laparoscopy represents a notable advancement in the treatment of advanced ovarian cancer (15). Additionally, biological and molecular characteristics play a critical role in selecting the most precise and personalized therapeutic strategies for patients (16). By evaluating prognostic factors that influence postoperative outcomes, clinicians can adopt targeted treatments to address the underlying causes of poor prognosis. Early detection and timely intervention remain essential for improving patients’ quality of life and extending survival. This study emphasizes the growing relevance of nutritional and inflammatory markers as significant predictors of postoperative prognosis in ovarian cancer patients. A deeper understanding of how these markers correlate with survival outcomes offers valuable insights for patient management and could inform the development of innovative therapeutic strategies.

An imbalance between inflammatory and anti-inflammatory processes can lead to cell necrosis, apoptosis, coagulation abnormalities, and immune suppression (17). NLR serves as a well-established marker of systemic inflammatory response, reflecting both the activation and regulation of inflammation within the body (18). Neutrophils play a pivotal role in this process by secreting pro-inflammatory cytokines, regulatory cytokines, and chemokines, which initiate an inflammatory cascade during infection. In contrast, lymphocytes, integral to the immune system, counteract inflammation by releasing anti-inflammatory factors such as interleukin-10 (IL-10) (19). Monocytes contribute significantly to the innate immune response, continuously secreting pro-inflammatory cytokines, enzymes, and growth factors upon recruitment, thus amplifying the inflammatory response. Clinical data analysis indicates that elevated NLR is associated with an increased risk of mortality. The RCS diagram further reveals that a higher LMR correlates with improved OS and CSS in postoperative ovarian cancer patients, whereas elevated NLR is linked to reduced OS and CSS.

Although NLR and LMR reflect the systemic inflammatory response resulting from immune activation, they fail to capture the immune-nutritional status, a critical factor influencing ovarian cancer prognosis. Immune and nutritional disturbances are integral to this prognosis. PNI, which assesses the nutritional and inflammatory state, is derived from serum albumin levels and lymphocyte counts. In contrast, NPS is a novel prognostic tool that combines NLR, LMR, albumin, and TC. Both PNI and NPS are simple to calculate and accurately represent the systemic nutritional, immune, and inflammatory status of patients (6, 9). PNI has been extensively used as a prognostic marker in various cancers, including colon, liver, and esophageal cancers. Furthermore, its clinical relevance has been demonstrated in non-cancerous conditions such as COVID-19, cardiovascular diseases, Crohn’s disease, and sepsis (20–22). However, the prognostic value of PNI in ovarian cancer remains inconsistent (23). For example, Yoshikawa et al. identified a PNI threshold of 46.5 for patients with ovarian clear cell carcinoma, showing that those in the high-PNI group had significantly better OS compared to the low-PNI group (24). This study identified significant associations between both PNI and NPS with OS and CSS in postoperative ovarian cancer patients. A PNI >51.2 was found to be a protective factor, correlating with improved survival outcomes. Patients with L-NPS demonstrated significantly better prognoses than those with H-NPS. RCS analysis further corroborated the relationship between higher PNI and extended OS and CSS. Subgroup analysis reinforced these findings, indicating that PNI is also linked to muscle loss during ovarian cancer treatment. Specifically, low PNI and reduced skeletal muscle mass independently predicted increased all-cause mortality (25). A separate investigation into preoperative PNI and OS in ovarian cancer patients reported significantly longer OS in the high-PNI group compared to the low-PNI group (26).

The role of nutritional inflammatory markers in postoperative prognosis for ovarian cancer is an important area of study, as demonstrated in this research. Prior studies established a scoring system—termed the peripheral blood score (PBS)—based on various peripheral blood parameters, including neutrophil count, lymphocyte count, monocyte count, albumin level, TC, and fibrinogen. This model revealed that lower PBS values were associated with better outcomes, while higher values were linked to poorer prognosis. Furthermore, PBS, along with FIGO grade and residual lesions, was identified as an independent predictor of OS and progression-free survival (PFS) in patients with epithelial ovarian cancer (EOC) (27). Our findings reinforce the notion that nutritional inflammatory markers are independent prognostic factors for OS and CSS in postoperative ovarian cancer patients. These results are consistent with previous studies (28), which highlight the significant influence of systemic inflammation and nutritional status on cancer prognosis (29). By analyzing a comprehensive cohort of clinical characteristics, laboratory parameters, and treatment modalities, this research addresses a notable gap in the current literature by establishing a direct link between nutritional inflammatory markers and survival outcomes in ovarian cancer patients. This study represents the first integration of nutritional inflammatory markers into predictive models for postoperative OS and CSS in ovarian cancer. Independent predictors for OS included age, PNI, NPS, tissue type, tumor size, targeted therapy, and diabetes, while those for CSS comprised age, NPS, tumor size, targeted therapy, and diabetes. Using these variables, nomograms were constructed that exhibited robust predictive accuracy for 3- and 5-year survival. However, calibration curves for 1-year survival revealed some discrepancies. The OS model demonstrated the highest predictive accuracy, with an AUC of 0.910, while the CSS model achieved an AUC of 0.844. External validation yielded AUC values of 0.898 for OS and 0.848 for CSS. This validation confirms the model’s generalizability, reliability, and clinical applicability. Although the external validation results showed slightly reduced efficacy compared to the initial cohort, the model still displayed strong predictive performance. The observed differences may be due to the smaller sample size in the external validation cohort and the population heterogeneity between the two hospitals. In conclusion, the prognostic nomogram incorporating PNI and NPS offers considerable diagnostic value for postoperative ovarian cancer patients.

The findings carry important implications for clinical practice. Identifying nutritional inflammatory markers as reliable prognostic indicators provides healthcare providers with a robust tool for stratifying patients according to their postoperative risk. This approach could inform more personalized treatment strategies, such as enhanced postoperative monitoring or targeted nutritional interventions to optimize inflammatory status both pre- and postoperatively. Additionally, the results support incorporating nutritional inflammatory markers into routine clinical assessments, which may improve the predictive accuracy of survival outcomes, thereby enabling more informed decision-making and enhancing the overall management of ovarian cancer patients.

This study has several limitations that warrant consideration. The retrospective design may have introduced bias, and the relatively small sample size could limit the generalizability of the results. Additionally, the research methodology is somewhat restricted due to the small sample size and the limited statistical approaches employed, which diminish methodological diversity. Despite data collection from two hospitals, the lack of broader diversity remains a concern. Future research should prioritize validating these results in larger, multicenter cohorts and exploring the underlying mechanisms by which nutritional and inflammatory status influence cancer progression and treatment response.

## 5 Conclusion

In summary, the nutritional inflammatory index serves as a significant independent prognostic factor for OS and CSS in ovarian cancer patients. Its integration into clinical practice may improve patient stratification and enable more tailored therapeutic strategies. The development of a nomogram based on key predictors highlights its potential value in clinical decision-making. Nevertheless, additional research is required to validate these results and investigate the mechanisms connecting nutritional inflammation to tumor dynamics and patient outcomes. A more comprehensive understanding of these interactions could improve prognostic models and optimize management approaches for ovarian cancer patients.

## Data Availability

The raw data supporting the conclusions of this article will be made available by the authors, without undue reservation.

## Supplementary material

Supplementary Figure 1 RCS diagram of the relationship between albumin, total cholesterol, CA125, CA199, CEA and HE4 and survival prognosis in patients with ovarian cancer after surgery.
