# Supplementary figures and images for: Relationship between nutritional-inflammatory markers and postoperative outcomes in ovarian cancer: a retrospective study
Source: Front Oncol. 2025 Mar 11;15:1531987. doi: 10.3389/fonc.2025.1531987 (PMC11932915; doi:10.3389/fonc.2025.1531987)

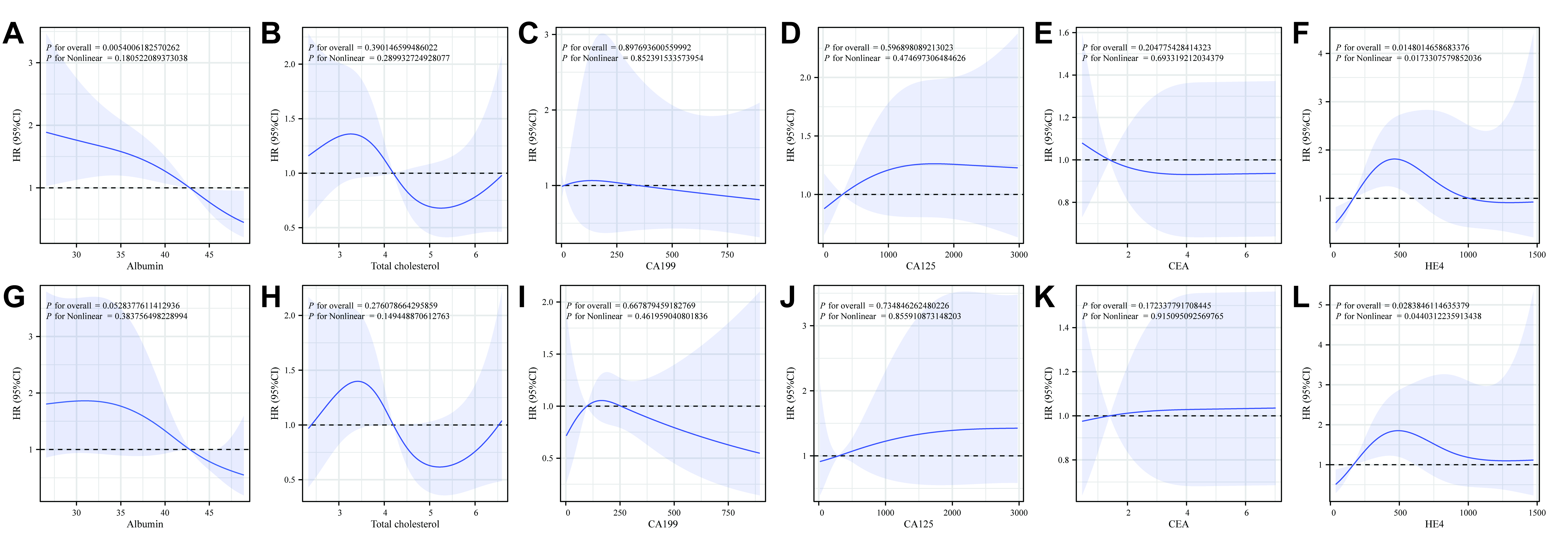

Supplement: Supplementary Figure 1 — RCS diagram of the relationship between albumin, total cholesterol, CA125, CA199, CEA and HE4 and survival prognosis in patients with ovarian cancer after surgery. [file Image1.tif]
